# *Symphyonema bifilamentata* sp. nov., the Right *Fischerella ambigua* 108b: Half a Decade of Research on Taxonomy and Bioactive Compounds in New Light

**DOI:** 10.3390/microorganisms9040745

**Published:** 2021-04-02

**Authors:** Patrick Jung, Paul M. D’Agostino, Burkhard Büdel, Michael Lakatos

**Affiliations:** 1 Applied Logistics and Polymer Sciences, University of Applied Sciences Kaiserslautern, Carl-Schurz-Str. 10-16, 66953 Pirmasens, Germany; Michael.lakatos@hs-kl.de; 2 Faculty of Chemistry and Food Chemistry, Technical University of Dresden, Chair of Technical Biochemistry, Bergstraße 66, 01069 Dresden, Germany; paul.dagostino@tu-dresden.de; 3 Biology Institute, University of Kaiserslautern, Erwin-Schrödinger Str. 52, 67663 Kaiserslautern, Germany; buedel@bio.uni-kl.de

**Keywords:** *Symphyonema*, true-branching, heterocytes, ambigol, polyphasic approach, *Fischerella*

## Abstract

Since 1965 a cyanobacterial strain termed ‘*Fischerella ambigua* 108b’ was the object of several studies investigating its potential as a resource for new bioactive compounds in several European institutes. Over decades these investigations uncovered several unique small molecules and their respective biosynthetic pathways, including the polychlorinated triphenyls of the ambigol family and the tjipanazoles. However, the true taxonomic character of the producing strain remained concealed until now. Applying a polyphasic approach considering the phylogenetic position based on the 16S rRNA and the protein coding gene *rbcLX*, secondary structures and morphological features, we present the strain ‘*Fischerella ambigua* 108b’ as *Symphyonema bifilamentata* sp. nov. 97.28. Although there is the type species (holotype) *S. sinense* C.-C. Jao 1944 there is no authentic living strain or material for genetic analyses for the genus *Symphyonema* available. Thus we suggest and provide an epitypification of *S. bifilamentata* sp. nov. 97.28 as a valid reference for the genus *Symphyonema*. Its affiliation to the family Symphyonemataceae sheds not only new light on this rare taxon but also on the classes of bioactive metabolites of these heterocytous and true-branching cyanobacteria which we report here. We show conclusively that the literature on the isolation of bioactive products from this organism provides further support for a clear distinction between the secondary metabolism of *Symphyonema bifilamentata* sp. nov. 97.28 compared to related and other taxa, pointing to the assignment of this organism into a separate genus.

## 1. Introduction

True-branching heterocytous cyanobacteria were formerly classified as Stigonematales [1], but various phylogenetic analyses have shown that the Nostocales form a monophyletic lineage within which the true-branching genera are scattered in several unrelated families, making Stigonematales polyphyletic [2,3] and thus an outdated taxon distinction. True-branching Nostocalean genera have the most complex morphological traits across the whole phylum Cyanobacteria, including various cell types, branching patterns, and thalli structures. Besides heterocytes (nitrogen fixation site) as the most distinctive cell type of all Nostocacean taxa, they can have akinetes (dormant resting cells), hormogonia (motile propagules), and necridia (controlled cell-death). Within the Nostocacean genera exhibiting false- or scytonematoid-branching pattern (e.g., *Scytonema*, *Brasilonema*), true-branching patterns of T-type (e.g., *Westiellopsis*, *Fischerella*), or Y-type (e.g., *Mastigocladopsis*, *Symphyonemopsis*) are included that can lead to a differentiated morphology of a basal section and significantly different lateral branches as commonly found in *Stigonema*, for example [2].

Although this wealth of striking morphological differences and characteristics that one might set into phylogenetic relations exists, it has been shown that true-branching cyanobacteria are polyphyletic based on genetics [2], and more recently based on heterocyte glycolipids [4]. According to the current taxonomic classification of cyanobacteria established by Komárek et al. in 2014 [3], the true-branched taxa are placed in the five families Symphyonemataceae (13 genera), Hapalosiphonaceae (28 genera), Stigonemataceae (5 genera), Capsosiraceae (4 genera) and Chlorogloeopsidaceae (1 genus). This makes a total of 51 genera of which only 11 (*Westiella*, *Spelaeonaias*, *Iphinoe*, *Loriellopsis*, *Chlorogloeopsis*, *Aetokthonos*, *Mastigocladus*, *Mastigocoleus*, *Westiellopsis*, *Neowestiellopsis*, *Reptodigitus*) are supported by molecular data such as the 16S rRNA and only a few of them comprising the holotype species. 

Resolving the taxonomy of true-branching cyanobacteria is complicated by the fact that only a few of these rare true-branching species have been deposited at public culture collections preventing a compiled resolution of their taxonomy in the context of novel, future isolates. In addition, most of them occur at extreme and thus less frequently sampled habitats such as thermal springs e.g., *Fischerella thermalis* [5], *Chlorogloeopsis* sp. [6], *Mastigocladus laminosus* [7], or caves e.g., *Spelaeonaias floccida* [8], *Geitleria calcarea* [9], *Symphyonema cavernicolum* [10]. In the case of the genus *Symphyonema,* a few species are described such as the aforementioned *S. cavernicolum* [10], *S. kabooruum* from the littoral zone of an acidic, coastal oligotrophic lake in Australia [11], the type species of the genus *S. sinense* [12] from wet, calcareous rocks of China and *S. sinense* var. *minor* from French Guiana [13] but without any of them being deposited at culture collections or available genetic information. The current genetic reference point for the whole genus are two 16S rRNA sequences of isolates from soil in Papua New Guinea that were not taxonomically defined but support the distinct phylogenetic position of this genus based on the 16S rRNA [2].

However, one of the true-branching strains that gained the attention of several researchers of various disciplines during the past is ‘*Fischerella ambigua* 108b’ (Hapalosiphonaceae) that was isolated in 1965 from wet soil in Switzerland. From that point forward, ‘*Fischerella ambigua* 108b’ was the subject of various studies on bioactive cyanobacterial products without confirmation of its taxonomic assignment. Between 1965 and 2020 several studies were conducted that led, among other things, to the discovery of the polychlorinated triphenyls ambigol A–E [14,15,16] and their biosynthetic pathway [17], and the isolation of the tjipanazoles and characterization of their biosynthesis [18]. These and other findings were always discussed in light of bioactive compounds and their metabolic pathways extracted from related species, such as *F. thermalis* or *F. muscicola*, or related genera, including *Westiellopsis*, *Hapalosiphon* or *Nostochopsis*. 

Here, we show that the strain ‘*Fischerella ambigua* 108b’, an important strain of many articles on drug discovery, in fact is a novel *Symphyonema* species based on a polyphasic approach. Using a combination of morphological information combined with the evaluation of its taxonomic position based on the 16S rRNA, 16S-23S ITS gene region, and the protein coding gene region *rbcLX* allowed the establishment of *Symphyonema bifilamentata* sp. nov. As there is no culture material or genetic information of any other *Symphyonema* species available, we suggest that *S. bifilamentata* sp. nov. should act as reference point for the genus along with an epitypification and emendation of the genus *Symphyonema* provided here. The holotype of the genus *Symphyonema* is *S. sinense* C.-C. Jao but refers to two plates in the original publication only [12]. However, there is no designated type material in the form of a preserved specimen or a type culture available. According to the discussion in [19], we believe that this justifies an epitypification and suggests the new species described here as a new type specimen for the genus. To account in the traceable history of science for this specific strain, we included a revision on its natural products discussed in the new light of its taxonomic position, which removes it from Hapalosiphonaceae and places it into the distantly related Symphyonemataceae.

## 2. Materials and Methods

### 2.1. Origin of Strain

Two identical cyanobacterial strains were isolated in 1965 from a shallow soil deepening that was wet at intervals at Mellingen, Switzerland, from Alfons Zehnder. Afterwards, both were given to the Swiss Federal Institute of Aquatic Science and Technology (EAWAG) culture collection (Dübendorf, Switzerland) as *Fischerella ambigua* 108a and 108b until they were transferred to the culture collection of Burkhard Büdel (University of Kaiserslautern, Department Plant Ecology and Systematics, Kaiserslautern, Germany) as strains 97.28a and 97.28b. In addition, strain 108b was also given to the Culture Collection of Algae and Protozoa (CCAP; Scotland) as strain 1427/4 in 2004. According to information from all culture collections the strains were cultured in liquid nutrient deficient standard medium for cyanobacteria BG11 and BG11_0_ (without nitrogen) medium at 17 °C, at photosynthetic photon flux density of 30 µmol m^−2^ s^−1^ and a light:dark cycle of 18:6 h, which were also the conditions during our investigations.

### 2.2. Morphological Characterization

The morphology of the cyanobacterial isolate was checked weekly over the course of several months by light microscopy using a Panthera KU Trinocular (Motic) equipped with 10×, 20×, 40× and 100× magnification and oil immersion coupled with a MicroLive Multi Format camera and the software MicroLive (v4.0). In addition, differential interference contrast (DIC) images were taken with an Axisokop (Carl Zeiss, Jena, Germany). This was carried out for cultures on solidified and liquid BG11 as well as BG11_0_ medium without nitrogen to enhance the growth of heterocytes [20]. Two hundred images were taken from the strain and the length and widths of the cells were measured for 50 cells with MicroLive (v4.0). Digital drawings were made with a touchpad tablet (Ugee M708) and Adobe Photoshop CS6 based on microscopic images.

### 2.3. DNA Extraction, Amplification and Sequencing

Genomic DNA of the strain *Fischerella ambigua* 108b/ CCAP1427/4/ 97.28 was extracted from unialgal cultures as described by Williams et al. [21]. Nucleotide sequences of the 16S rRNA gene together with the 16S–23S ITS region (1700–2300 bases) were amplified as described by Marin et al. [22] using the primers SSU-4-forw and ptLSU C-D-rev. The *rbcLX* gene region (1200 bases) was amplified using the primers rbcLX-CW and rbcLX-CX as described by Rudi et al. [23]. 

The quality of the polymerase chain reaction (PCR) products were checked by means of agarose gel electrophoresis using 1% (*w*/*v*) agarose and subsequently purified with the NucleSpin Gel and PCR Clean-up Kit (Macherey-Nagel GmbH & Co. KG, Düren, Germany) following the DNA and PCR clean up protocol. The purified PCR products were sent to Genewiz (Germany GmbH, Leipzig, Germany) for Sanger sequencing with the primers SSU-4-for, Wil 6, Wil 12, Wil 14, Wil 5, Wil 9, Wil16, and ptLSU C-D-rev [22,24,25] for the 16S rRNA and rbclX-CW and rbcLX-CX for the protein coding gene region [23]. The generated sequences were assembled with Geneious Prime (v2021.0.1) software package (Biomatters Limited, New Zealand). The sequences were submitted to the National Center for Biotechnology Information (NCBI) GenBank as stated in the species description.

### 2.4. Molecular Characterization

The assembled 16S rRNA and *rbcLX* gene sequences obtained from *Fischerella ambigua* 108b/ CCAP1427/4/ 97.28 and related sequences of cyanobacterial strains cited from GenBank were used for phylogenetic analyses including *Gloeobacter violacaeus* as outgroup for the 16S rRNA alignment and *Synechocystis* as outgroup for the *rbcLX* alignment. Both alignments were prepared applying the Muscle algorithm in Mega X [26]. 

Finally, 89 nucleotide sequences were used for the phylogenetic comparison including 1502 bp of the 16S rRNA gene as well as 57 nucleotide sequences for the *rbcLX* calculations including 782 bp characters. Ambiguous regions within each alignment were adjusted or removed manually allowing smaller final blocks and gap positions within the final blocks. The evolutionary model that was best suited to the used database was selected on the basis of the lowest AIC value and calculated in Mega X for both gene regions. The phylogenetic tree for the 16S rRNA was finally constructed with Mega X using the evolutionary model RGT+G+I of nucleotide substitutions for the alignment as well as T92+G+I for the phylogenetic tree of the *rbcLX* gene region. The maximum likelihood method (ML) with 1000 bootstrap replications was calculated with Mega X and Bayesian phylogenetic analyses with two runs of eight Markov chains were executed for one million generations with default parameters with Mr. Bayes 3.2.1 [27] for both trees. Each analysis reached stationarity (average standard deviation of split frequencies between runs < 0.01) well before the end of the run.

Models of the secondary structure of 16S–23S ITS region of *Fischerella ambigua* 108b/ CCAP1427/4/ 97.28 were built in comparison to phylogenetic or morphologically related genera such as *Fischerella*, *Brasilonema* and *Scytonema* according to the models proposed in Wilde et al. [28], Romanenko et al. [29] and Johansen et al. [30]. The secondary structures could not be compared to other *Symphyonema* species as the genetic information of these gene regions does not exist [2]. Helices were folded with the online software RNAstructure Web Server [31].

### 2.5. Holotype Preparation

The species was described following the rules and requirements of the International Code of Nomenclature for algae, fungi, and plants [32]. Furthermore, young (3-week-old) cultures were preserved in 4 % (*v*/*v*) formaldehyde, in 15 mL glass bottles. Preserved material was then deposited in the Herbarium Hamburgense, Hamburg, Germany (HBG-024930). 

## 3. Results

The strain originally termed *Fischerella ambigua* 108b/ CCAP1427/4/ 97.28 was found to be unique based on its ecology, morphology, distribution, phylogeny and secondary structures of the 16S-23S ITS gene region and not related to *Fischerella ambigua*. Instead it showed high relation to the genus *Symphyonema* but because the combination of diacritical features associated with this species did not correspond with any described species within the genus, we named the strain here as the new species *Symphyonema bifilamentata* sp. nov. 97.28. The most striking morphological characteristic of *S. bifilamentata* sp. nov. 97.28 is its clear separation between basal section and lateral branches what is explicitly excluded in the original description of the genus *Symphyonema* by C.-C. Jao in 1944 and thus requires an emendation of the genus.

### 3.1. Taxonomic treatment

#### 3.1.1. *Symphyonema* C-C. Jao 1944; Emend. P. Jung, B. Buedel et M. Lakatos

Emended diagnosis: Thallus pulvinate or wooly, up to 1.5 cm thick, greyish blue with multiple densely coiled, or parallel-arranged filaments, sometimes joined into erect fascicles. Filaments irregularly branched, sometimes distinctly morphologically diversified in basal sections and branches. Trichomes uniseriate, isopolar, with vegetative cells usually longer than wide, cylindrical, not constricted or slightly constricted at cross walls, not attenuated towards the ends, with rounded terminal cells. Cell content blue-green, often with granulation of various graininess and/or with prominent vacuole-like rounded structures. Branching of two types, true branching of Y-type (reverse Y-branching originates always from special oblong cells within basal sections, each followed by two shortened cells, from which one divides into one elongated cell that starts the lateral branch) and/or T-type (initiates at two swollen, globose cells with prominent, vacuole-like and granular inclusions that divide into smaller, rounded to isodiametric cells that start the lateral branch) and false-branching of scytonematoid-type (including necridic cells); branching initiates remote from the heterocytes. Sheaths firm, homogeneous or slightly lamellated, yellow-brown in color in mature parts. Vegetative cells blue-green in color with slightly granular content. Heterocytes intercalary, cylindrical, solitary. Akinetes not observed. Cell division crosswise to the trichome length. Reproduction mostly by hormogonia, separating from trichome apices or defragmentation of, especially, the lateral branches. A small genus with three species currently taxonomically accepted: *S. cavernicolum* from a cave in Spain [10], *S. kaboorum* from the littoral zone of an acidic, coastal oligotrophic lake in Australia [11], the former type species of the genus *S. sinense* [12] from wet, calcareous rocks of China and *S. sinense* var. *minor* from Papua New Guinea [13] but without any of them being deposited at culture collections nor available genetic information. Two sequenced isolates from soil in Papua New Guiana that were not taxonomically defined supported the distinct phylogenetic position of this genus [2].

Comments: originally the holotype for the genus *Symphyonema* was *S. sinense* Jao C.-C. but there is no culture or preserved material nor genetic information available. As *S. bifilamentata* closely conforms to descriptions for all species described for the genus we propose *S. bifilamentata* as new reference for the genus *Symphyonema.* Following the rules for epitypification established in the International Code for Algae, Fungi and Plants [32] and using the monophyletic species concept, we formally present the epitypification.

Epitype for the genus *Symphyonema* designated here: HBG-024930, culture material of *Symphyonema bifilamentata* 97.28 (DSM 112338) preserved in 4 % formaldehyde, Herbarium Hamburgense, Hamburg, Germany.

Epitype strain: *Symphyonema bifilamentata* 97.28 is available at the culture collection DSMZ Braunschweig, Germany (DSM 112338).

#### 3.1.2. *Symphyonema bifilamentata* sp. nov. P. Jung, B. Buedel et M. Lakatos 

**Figure 1:**
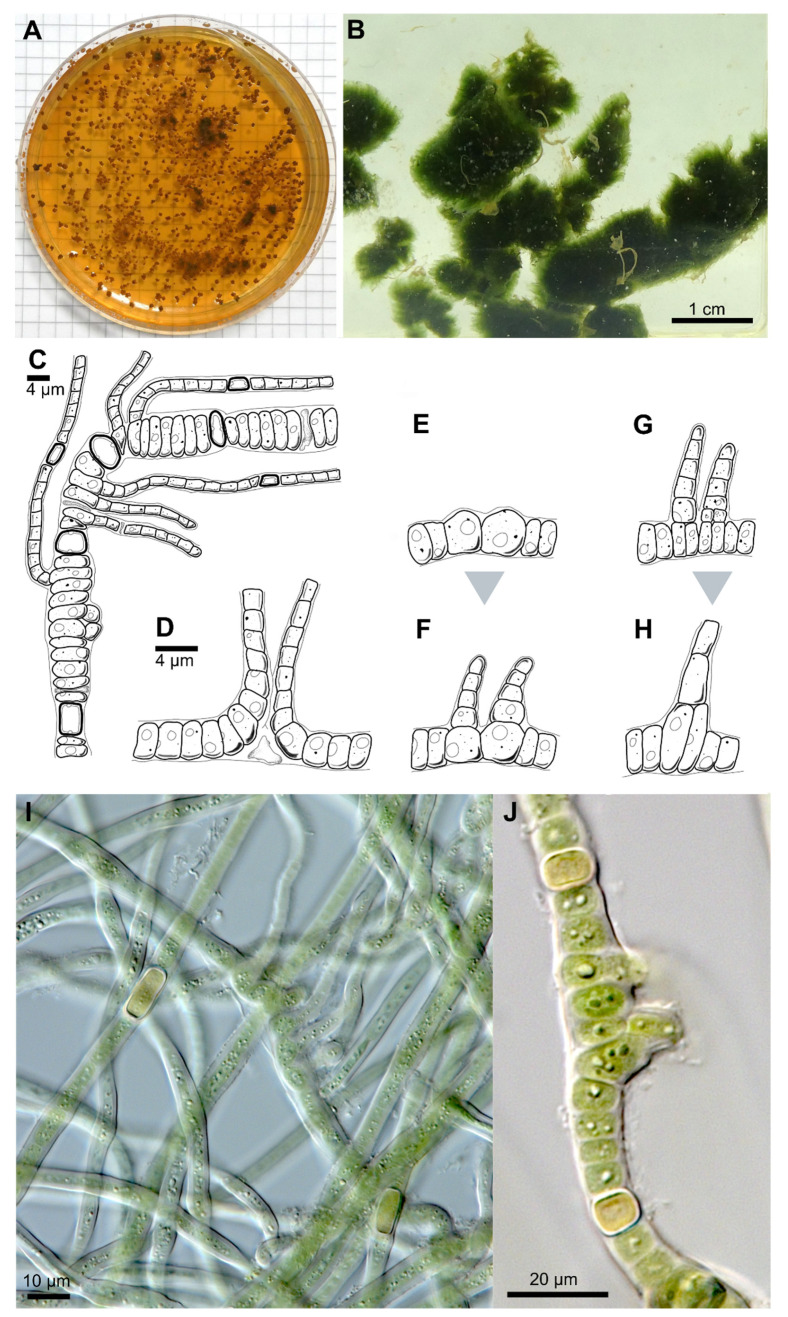
Photographs and drawing of *Symphyonema bifilamentata*. (**A**) One-year-old culture on standard BG11 agar with segregated brownish to orange substance that stained the medium. (**B**) Photograph of mature thallus-like culture in liquid BG11 with fresh medium. (**C)** Overview drawing of a mature filament showing bloated basal sections with several lateral branches. (**D**) Scytonematoid type of false branching of young basal section with necridic cell. (**E**,**F**) Development of true T-branching with initial swollen basal cells within young basal section in (**E**) and trapezoidal cells on each basal cells that extend to lateral branches in (**F**). (**G**,**H**) True Y-branching of basal sections with initial stage in (**G**) and mature filament in (**H**–**J**) show DIC micrographs of mature culture with wider sheaths, multiple branchings and heterocytes.

**Figure 2:**
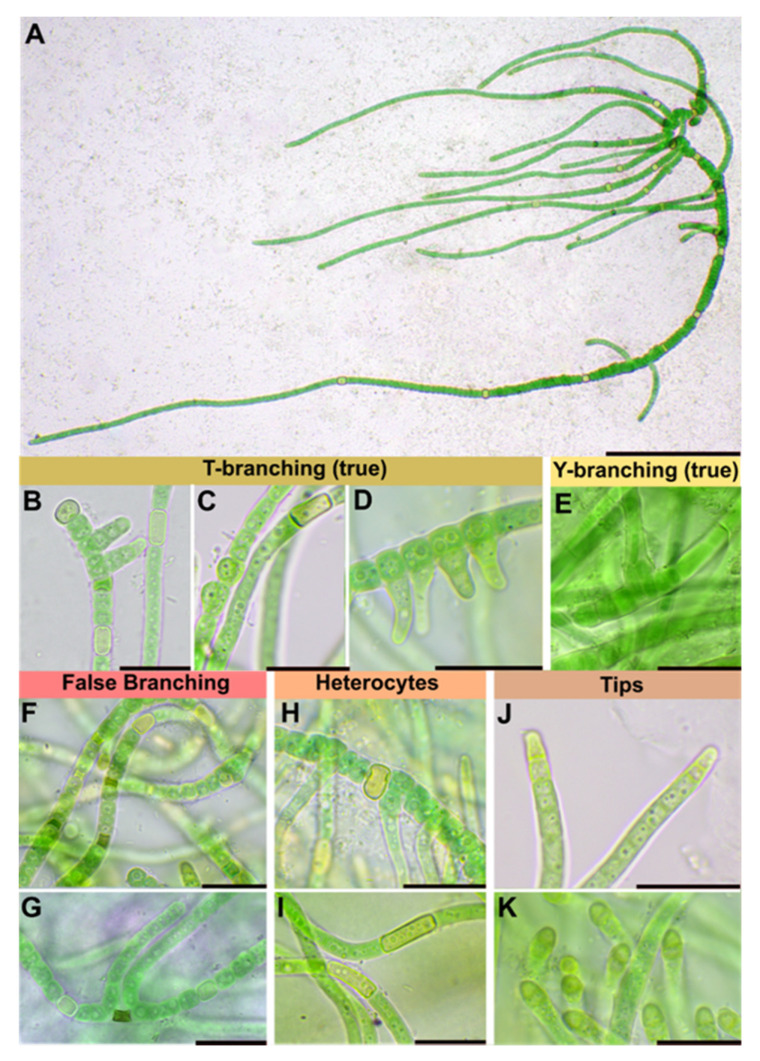
Morphological details of *Symphyonema bifilamentata* sp. nov. (**A**) Overview image showing a mature filament growing on agar with swollen basal section that is tapered at heterocytes and several bilateral branches with heterocytes. Scale bar 50 µm. (**B**–**D**) Formation of true T-branching out of young basal sections. € True Y-branching with two elongated cells that unite to a lateral section. (**F**–**G**) Necridic cells in young basal sections in (**F**) and scytonematoid false branching in (**G**) where both cells surround a necridic cell form lateral filaments that grow out of the sheath. (**H**) Squeezed globose heterocyte of basal section. (**I**) Elongated heterocytes of lateral branches. (**J**) Yellowish apical cells of young lateral branches and (**K**) indicating yellowish rounded tips of mature lateral branches. Note fine granulation of lateral sections in (**I**–**K**) compared to the coarse vacuole-like granulation of basal sections in (**B**–**D**) and (**H**). Scale bar of (**B**–**K**) indicates 10 µm.

Description: Pulvinate macroscopic thallus, up to 1 cm thick, dark green, wooly, that dyes liquid medium transparent-brownish during aging. Filaments are interwoven, on agar first creeping, later slightly erect and parallel arranged. Two types of filaments can be distinguished from each other, swollen basal branches and thin lateral branches. Basal branches: trichomes 3.3–6.6 µm wide, with necridia, mostly uniseriate, bloated but tapered/waisted towards heterocytes that were always present in medium with and without nitrogen; cells barrel-shaped, rounded, squeezed, constricted at cross-walls, wider than long, 2.4–3.3 (6) µm × 1.3–1.7 µm, coarse granulated, with at least one big vacuole-like structure in each cell, blue-green. Lateral branches: up to 200 µm long, uniseriate, without necridia; filaments straight, parallel; regular, not tapered towards the ends, fine granulated, fine vacuolated, cells regular, quadratic or slightly longer than wide, 2–2.3 µm × 2–2.8 µm, very slightly constricted at cross-walls, blue-green; apical tip rounded to slightly conical, yellowish, 2.8 µm × 1.8 µm. Branching patterns are of true-branching T-type towards all sides of the basal section and false-branching (scytonematoid). Heterocytes are frequent independent of the culture medium used which occur solitary or rarely in pairs of two. They are thick-walled, yellow, intercalary and rectangular in lateral branches, 2.3 µm × 3.6–5 (8.5) µm but sub-globose, squeezed and bloated in basal sections, 2–2.2 µm × 3.6–3.9 µm. Sheaths of the trichomes are hyaline, colorless, firm, limited, not lamellated, very tight but ruptured by the apical cells of lateral branches. Mature cultures with limited, hyaline but much wider sheaths. Hormogonia or akinetes were not observed, fragmentation of lateral branches (tips) as main dispersal pattern (Figure 1 and Figure 2; Table 1).

Habitat: shallow soil deepening that was every then and now wet at Mellingen, Switzerland.

Etymology: ‘*bifilamentata*’—‘having two types of filaments’, due to the clear morphological separation of the species into basal and lateral sections.

Type location: SWITZERLAND—Mellingen, Switzerland isolated by Alfons Zehnder in 1965.

Holotype: The preserved holotype specimen (strain 97.28) is available via the Herbarium Hamburgense, Hamburg, Germany (HBG-024930). It was prepared from the living strain which was the source of 16S, ITS, and 23S rRNA gene sequence (GenBank accession number MW565974) and rbcLX (GenBank accession number MW565964).

Reference strain: The reference strain *Symphyonema bifilamentata* sp. nov. 97.28 is available at the German Collection of Microorganisms and Cell Cultures GmbH (DSMZ) Braunschweig, Germany (DSM 112338). Access is also given via the Culture Collection of Algae and Protozoa (CCAP), Scotland (1427/4) since 2004.

Discrimination against other species: *Symphyonema bifilamentata* sp. nov. has overall smaller trichomes and cells than *S. sinense*, *S. cavernicolum* and *S. kaboorum* and basal and lateral branches are strictly divaricated and frequently shows heterocytes. Additionally, the basal sections of *S. bifilamentata* sp. nov. are bloated and tapered towards heterocytes.

**Figure 3:**
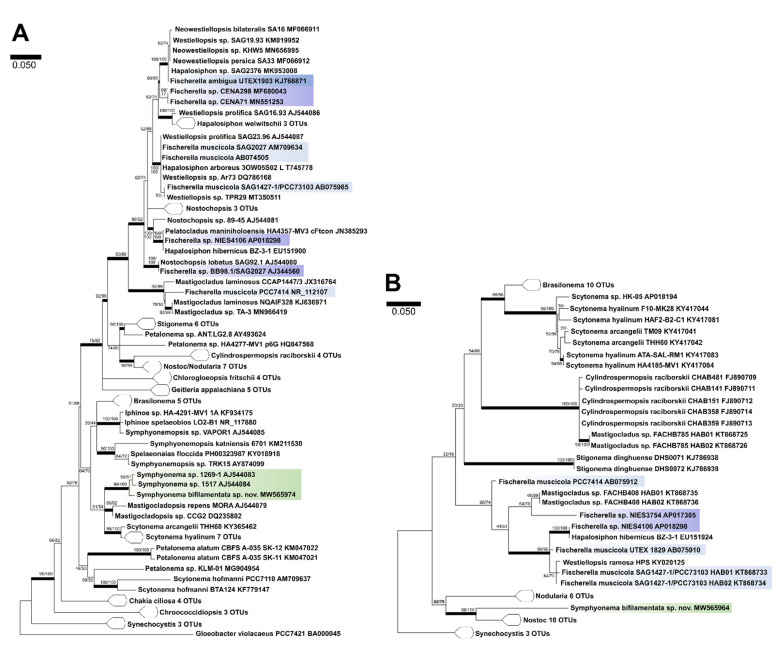
Comparative phylogenetic trees of 16S rRNA and protein coding gene region *rbcLX*. (**A**) Maximum likelihood (ML) tree obtained from 89 aligned 16S rRNA gene sequences with 1508 bp indicating the position of *Symphyonema bifilamentata* sp. nov. 97.28 rooted to *Gloeobacter violacaeus* PCC7421. (**B**) ML tree from 57 aligned sequences of the protein coding gene region rbcLX with 782 bp rooted to *Synechocystis*. Numbers on the nodes represent ML- and Bayesian bootstrap values respectively, (1000 replicates, each). Bold horizontal lines show lineages supported by at least 75 % of both bootstrap values. Bar represents 0.05 substitutions per nucleotide position. *Symphyonema* clade is highlighted in green, strains assigned to *Fischerella ambigua* in dark blue, strains assigned to *F. muscicola* are marked in light blue and *Fischerella* strains unassigned to species level are highlighted in purple.

**Figure 4:**
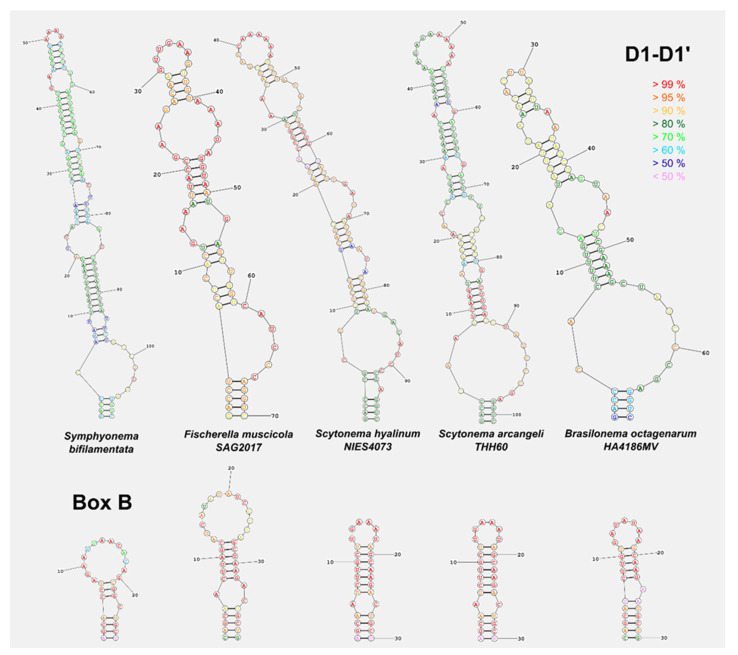
Predicted secondary RNA structures of the main informative helices of the 16S-23S ITS region (D1-D1′ and Box B) of *Symphyonema bifilamentata* sp. nov. 97.28 in comparison with the closest strains in terms of phylogenetic position and morphology. Color code indicates the probability in percent of the calculated base position.

Phylogenetic relations and secondary structure of the 16S-23S ITS sequence: Based on the 16S rRNA *Symphyonema bifilamentata* sp. nov. 97.28 clusters together with *Symphyonema* sp. 1296-1 and *Symphyonema* sp. 1517 from soil in Papua New Guinea together with the genus *Mastigocladopsis* and the *Scytonema hyalinum* complex Figure 3A. The species is most closely related to *Symphyonema* sp. 1296-1 (98.73 %), *Symphyonema* sp. 1517 (98.41 %) and *Syctonema hyalinum* FI5-JRJ03 clone 13 (96.13 %). Secondary structure of the main informative helices of 16S–23S ITS sequences shows significant differences (Figure 4) in the D1-D1′ region and Box B compared to morphological or phylogenetically related taxa such as *Fischerella*, *Brasilonema*, and *Scytonema*. Loops of the D1-D1′ and Box B of *S. bifilamentata* sp. nov. 97.28 are on different positions than those of e.g., *Fischerella muscicola* SAG 2017, and the Box B sequence is shorter than those of the other strains. Secondary structures of other Symphyonemataceae could not be created because no other representative is available in the culture collection nor do there exist molecular data of the corresponding gene regions.

## 4. Discussion

### 4.1. Taxonomic Significance

The Symphyonemataceae appear to be an interesting family comprising 11 genera of true-branching heterocytous cyanobacteria of which only a few species were studied using molecular methods including data of the 16S rRNA gene of the type species [3]. Surprisingly, this still holds true although a lot has changed in the systematics of cyanobacteria throughout the last decades fueled by modern sequencing techniques and a plethora of studies that include isolation of strains. 

In addition, for the genus *Symphyonema* there is no genetic information from neither the type species *S. sinense* [12], nor for any of the described species such as *S. kaboorum* [11], *S. cavernicolum* [10] or *S. cavernicolum* var. *minor* [13] available. However, the previous authors described their findings well based on morphological features which allows a presumably correct assignment of the species to this genus and discrimination among those species. From a holistic point of view, the two isolates *Sympyhonema* sp. 1269-1 and 1517 from soil of Papua New Guinea [2] acted as well-supported entities that bridged the gap between the morphological establishment of the genus and genetically captured strains. However, the picture of the genus *Symphyonema* was still incomplete since the two strains were not deposited at any culture collection and thus cannot be evaluated in the context of the exacting, rule-bound taxonomic agreement. Finally, this work establishes a valid fixpoint of the genus with *S. bifilamentata* sp. nov. 97.28 as an isolate of known origin, trackable history, morphological evaluation, poly-genetic analyses and granted access via several culture collections. 

In contrast to its original morphology-based assignment to *Fischerella ambigua* in 1965, the strain clustered distantly from the family Hapalosiphonaceae including several species of *Fischerella* in the ML tree of the 16S rRNA (Figure 3A), but appeared in close relation to sequences of *Symphyonema* sp. 1269-1 and 1517, with >98 % similarity. Interestingly, the three *Symphyonema* sequences form a bigger cluster (Figure 3A) together with the true-branching *Mastigocladopsis*, and the false branching *Scytonema hyalinum* complex (<96% similarity) that again demonstrates the polyphyly of true-branching heterocytous taxa, formerly classified as Stigonematales. The protein coding gene region *rbcLX* of *Symphyonema bifilamentata* sp. nov. 97.28 shows a single position of the sequence in the ML tree but here it clusters together with the genera *Nostoc* and *Nodularia* (Figure 3B). This appears confusing but it needs to be mentioned that the nucleotide data bases currently hold only a limited amount of sequences of this gene region for comparisons. Information about this gene region from highly related taxa such as *Mastigocladopsis* are missing which explains the single position of the *rbcLX* sequence of our strain. However, this gene region is proved to be a significant discrimination feature especially within the Nostocales because it is a single copy gene of approximately 1430 base pairs which is free from length mutations except at the far 3′ end with a fairly conservative rate of evolution [33]. 

The separation from other *Symphyonema* species can also be supported by examination of morphological features. For example, the prominent differentiation into main and lateral branches (Figure 2A) together with the bloated parts of the main sections tapered by heterocytes (Figure 1C; Figure 2A) allows a strict separation (Table 1) from other *Symphyonema* species. However, this separation is also supported by the different ecology of the species, regarding the studied strain, collected from soil in Switzerland where it survives temperatures below 0 °C during winter. While the other species are from rather ice free areas such as from granitic rocks in French Guiana (*S. sinense* var. *minor*), low-light cave in Spain (*S. cavernicolum*), calcareous rocks in China (*S. sinense*) and an acidic, coastal oligotrophic lake in Australia (*S. kaboorum*). 

The initial description of the strain 108b as *Fischerella ambigua* was on the one hand probably misguided by the fact that the locality of the type strain of *F. ambigua* was Zurich, Switzerland [34] and the location of the strain 108b was also based in Switzerland, only ca. 30 km away at Mellingen. With the knowledge about cyanobacteria at the time strain 108b was found, it was much more likely that it resembled a cyanobacterial species from the same sampling area than a species that was once found in Asia. On the other hand *F. ambigua* and *S. bifilamentata* share some basic morphological features but can be distinguished based on e.g., the multiseriate filaments found in *F. ambigua* that were part of the first description [34] and investigated elsewhere [35]. In 1965 the situation might have also been complicated by the fact that the description of *Symphyonema* was published in an Asian magazine [12] while literature about *F. ambigua* was available in French [34], which was much more accessible to the Swiss isolator.

### 4.2. Bioactive Compound Biosynthesis: Symphyonema vs. Fischerella

**Figure 5:**
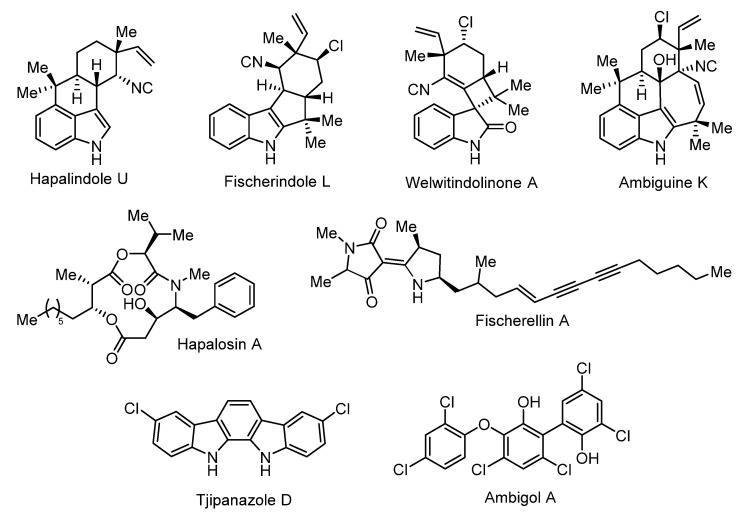
Representative chemical structures from *Symphyonema bifilamentata* sp. nov. 97.28 and various strains of *Fischerella*.

The true-branching cyanobacterial genera in the Nostocales were first investigated for their production of novel natural products in the mid-1980s, mainly spurred by the work of Prof. Richard E. Moore [36]. The biosynthetic potential of these organisms has since also been observed at the genome level, with large numbers of biosynthetic gene clusters being identified throughout the order, despite the limited number of sequenced genomes [37]. Within the true-branching genera of the Nostocales, the majority of cyanobacterial isolates belong to the genus *Fischerella* and these provide a significant proportion of structurally intriguing bioactive products limited to this order (Figure 5; Table 2). Interestingly, while the majority of true-branching genera of the Nostocales have been isolated from freshwater or terrestrial environments, they appear to produce an extraordinary array of halogenated bioactive products. The presence of halogen substituents often enhances the bioactivity of these products [38] and, therefore, the true-branching Nostocales, especially *Fischerella*, have been greatly focused upon in natural product discovery efforts.

By far the largest group of bioactive products reported from the *Fischerella* genus are the hapalindole-like family of indole alkaloids [39], which consist of more than 80 analogues divided into four subgroups, including the hapalindoles, fischerindoles, ambiguines, and the welwitindoliones, each with characteristic oxidation and cyclisation patterns (Figure 5 for representative examples). The hapalindole-like family is dispersed throughout the true-branching genera of Nostocales including *Hapalosiphon*, *Westiella*, *Westiellopsis*, and most commonly *Fischerella*. Only one other class of bioactive products, the hapalosins, have been reported across two different genera within the Nostocales, initially reported from *Hapalosiphon welwitischii* IC-52-3 [37,40] and more recently in *Fischerella* sp. PCC9431 [41,42]. Two groups of bioactive products, the ultraviolet (UV) sunscreen mycosporine-like amino acids and the hepatotoxic microcystins, are broadly distributed across the cyanobacterial phylum including *Fischerella* [43,44,45]. Several other bioactive compounds including the fischerellins [46,47] parsiguine, and the aranazoles [48,49] appear to be strictly limited to production by *Fischerella*. Importantly, all of the natural products described within the *Fischerella* genus do not appear to be produced by *Symphyonema bifilamentata* sp. nov. 97.28.

The ambigols, 2,4-dichlorobenzoic acid and the tjipanazoles were originally reported from extracts of *Fischerella ambigua* 108b (now *Symphyonema bifilamentata* spec. nov. 97.28) in the early 90s and mid-2000s [14,15,50] and recently also accessed by total synthesis [51]. The ambigols are polyhalogenated triphenols that are composed of dechlorinated phenol building blocks are linked by biaryl or biaryl ether bonds. While they are structurally related to the polybrominated diphenyls recently reported from marine cyanobacteria [52,53], they remain biosynthetically unrelated [17]. Furthermore, the ambigols were reported to possess a range of bioactivities including antifungal, antibacterial and molluscicidal, as well as having been implicated in the increase of the quorum-sensing regulated red pigment prodigiosin. The tjipanazoles are halogenated bis-indole alkaloids with some structural similarities to the bioactive staurosporines from Streptomyces [54]. Several tjipanazoles were observed to have in vitro antifungal activity as well as weak cytotoxicity. With the only exception of the identification of several tjipanazole analogues from *Tolypothrix tjipanasensis* [55], all three groups of molecules have exclusively been identified in *Fischerella ambigua* 108b (*Symphyonema bifilamentata* sp. nov. 97.28). 

Since the initial discovery of the ambigols and tjipanazoles, these molecules have not been discovered in any other *Fischerella* strain. The biosynthetic gene cluster for both the ambigols (*ab*) and tjipanazoles (*tjp*) were recently characterised by Duell et al., [17] and Chilczuk et al., [18], respectively. To determine if other organisms may have the genetic capability to produce these molecules, we performed a bioinformatic screening across all genomes within the NCBI database using cblaster [56]. This revealed that currently the *ab* or *tpj* gene clusters cannot be found in any organism other than *Symphyonema bifilamentata* sp. nov. 97.28, indicating the biosynthetic gene clusters are not present in the currently available genomes of *Fischerella*. This finding highlights that the chances of discovering novel metabolites are greater by examining strains from new genera rather than additional representatives within the same genus, an idea that was recently supported also for other bacteria [57]. These results, in combination with the natural products isolation literature presented here (Table 2), provide support for a clear distinction between the secondary metabolism of *Fischerella* and *Symphyonema bifilamentata* sp. nov. 97.28 further highlighting the allocation of this organism into a separate genus. 

**Table 2:** Summary of natural products produced by *Symphyonema bifilamentata* 97.28 compared to those produced by related species from literature.

Compound Family	Producing Organism	Natural Product Class	Proposed Bioactivity	Reference
Ambigols	***Symphyonema bifilamentata*** **97.28**	Polyhalogenated aromatics	Antibiotic, antifungal, molluscicidal	[14,15,16,17,58]
2,4-dichlorobenzoic acid	***Symphyonema bifilamentata*** **97.28**			[15]
Tjipanazoles	***Symphyonema bifilamentata*** **97.28** *Tolypothrix tjipanasensis*	indolo[2,3-a]carbazoles	Antifungal	[18,55,59]
Aranazoles	*Fischerella* sp. PCC 9339	Halogenated NRPS/PKS		[49]
Fischerellins	*Fischerella muscicola*	Aminoacylpolyketide	Antifungal, herbicidal	[46,47,60,61]
	*Westiellopsis* sp. SAG 20.93			
Ambiguines	*Fischerella* sp. *Fischerella ambigua* *Fischerella ambigua* UTEX 1903 *Hapalosiphon* sp. *Hapalosiphon delicatulus* *Hapalosiphon hibemicus* BZ-3-1 *Westiellopsis prolifica* EN-3-1	Halogenated Indole alkaloid	Antimicrobial, phytotoxic	[62,63,64,65,66,67]
Fischerindoles	*Fischerella sp.* SAG 46.79 *Fischerella muscicola* *Fischerella muscicola* UTEX 1829	Halogenated Indole alkaloids	Antifungal	[60,68]
Hapalindoles	*Fischerella ambigua* UTEX 1903 *Fischerella* sp. *Fischerella* sp. CENA 19 *Fischerella muscicola* UTEX LB1829 *Westiellopsis* sp. SAG 20.93	Halogenated Indole alkaloids	Insecticidal	[61,66,69,70,71,72]
Welwitindolinones	*Fischerella muscicola* HG-39-5 *Fischerella major* HX-7-4 *Hapalosiphon welwitschii* IC-52-3 *Westiella intricata* HT-29-1	Halogenated Indole alkaloids		[40,73]
Hapalindolinones	*Fischerella* sp. ATCC 52558	Indole alkaloids		[74]
13-hydroxydechlorofontonamide	*Fischerella muscicola* UTEX LB1829	Indole alkaloids		[61]
Mycosporine-like amino acids	*Fischerella* sp. PCC9339 *Fischerella* sp. 9431			[43]
Microcystins	*Fischerella* sp. NQAIF311 *Fischerella* sp. CENA161 *Fischerella major*	NRPS/PKS	Hapatotoxin	[44,45,75,76]
Parsiguine	*Fischerella ambigua* PTCC 1635		Antimicrobial	[48]
Hapalosin	*Hapalosiphon welwitschii* IC-52-3 *Fischerella* sp. PCC9431	NRPS/PKS	Multidrug-resistance reversing activity	[37,40,41,42]

NRPS = Non-Ribosomal Peptide Synthetase; PKS = Polyketide Synthase.

## 5. Conclusions

Our study demonstrates the importance of combining classical taxonomical work in order to correctly interpret the identification of natural products such as bioactive compounds produced by microalgae and their potential applications. This includes the whole workflow from isolating, evaluating ecological factors from the original environment, taxonomic work ranging from classical morphology to genetics, as well as the deposition of authentic strains in public culture collections. Moreover, the combination of these and biochemical methods not only help to separate taxa, but also indicate the probability to find new metabolic pathways and thus compound families. The authors want to encourage also those who conduct studies on cyanobacterial strains to carefully evaluate the taxonomic character of their strains, because this allows a correct assignment of results in comparison to related strains, genera or species.

## Figures and Tables

**Table 1:** Morphological comparison of *Symphyonema* species including *Fischerella ambigua*.

	*Symphyonema sinense* C.-C. Jao	*Symphyonema sinense var. minor C.-C.* Jao	*Symphyonema cavernicolum* Asencio, Aboal et Hoffmann	*Symphyonema kaboorum* G. B. McGregor	*Symphyonema bifilamentata* sp. nov. P. Jung, B. Buedel et M. Lakatos	*Fischerella ambigua* Kützing ex Bornet et Flahault) Gomont	*Symphyonema* sp. 1269-1	*Symphyonema* sp. 1517
Origin	Aerophytic, wet calcareous rocks, Kwangsi, China; Jao 1944	Epilithic (subaerial), granite inselberg French Guiana; crest of Montagnes Balenfois (N 4°5′, W 52°42′) 1989; Sarthou et al., 1995	Epilithic/chasmoendolithic, limestone cave, karstic region, Los Almadenes gorge, Spain; Asencio et al., 1996	Aquatic; surface of large, submerged Logs/woody debris in the littoral zone of an acidic, coastal oligotrophic lake, Naree Budjong Djara National Park, Stradbroke Island, Queensland, Australia; McGregor 2018	Edaphic, shallow, hollow, wet soil, Mellingen, Switzerland, isolated 1965; this work	Edaphic, wet, unpolluted soil, among mosses; Zurich; Gomont 1895	Edaphic, soil, Papua New Guinea, isolated 1986; Gugger and Hoffmann, 2004	Edaphic, soil, Papua New Guinea, isolated 1986; Gugger and Hoffmann, 2004
Thallus	Pulvinate, later expanded, up to 1.5 cm thick, greyish-blue; filaments parallel arranged, mostly erect,		Greyish, interwoven filaments	Pulvinate, formed by more or less parallel arranged filaments	Pulvinate, up to 1 cm thick, dark green, wooly; interwoven filaments, on agar mainly creeping, parallel arranged rarely slightly erect; dyes liquid medium transparent-brownish	Prostrate, dark brown, or only in in solitary filaments	Creeping filaments	Creeping and erect filaments
Branching Type	False-branching (scytonematoid), usually solitary, less frequently geminate	False branching, true-branching, reverse-type true branching	Mainly Y-type, but also T-type and false-branching (scytonematoid)	T- and Y-type true branching, false-branching (scytonematoid)	T-type true-branching towards all sides of the basal section, false-branching (scytonematoid)	T- and Y-type true branching, false-branching (scytonematoid)	Mainly Y-type but also T-type true-branching and false-branching (scytonematoid)	Mainly Y-type but also T-type true-branching and false-branching (scytonematoid)
Basal/main section	Filaments 9–12 µm wide, flexuose, irregularly ramified, densely intricate; trichomes 7–10 µm wide, commonly not constricted at cross walls; cells cylindrical, 15–38 µm long, up to 1.5, up to 3× longer than wide; blue green; apical cells rounded	Filaments 8–13 µm without ramification, simple; cell content homogeneous, greenish, cells cylindrical (6)8–14 µm × 2–4(5) µm	No differentiation; trichomes uniseriate, constricted at cross-walls, often tapering towards ends; cells irregular barrel shaped or cylindrical, isodiametric or longer than wide, 4–9.6 µm × 4–8 µm; pale blue green to violet, with scattered cyanophycin granules	Filaments 11–17 μm wide, erect, straight to irregularly flexuous, not tapered towards the ends, irregularly ramified, densely intricate, not constricted at the cross walls; Vegetative cells isodiametric, or up to 4 × longer than broad, shorter than broad towards the apices, (8.5–) 12.5–18.5 (–26) μm long × 4.8–10.5 μm wide, with fine granular contents, often vacuolate; apical cells rounded	Strictly divaricated from lateral branches; trichomes 3.3–6.6 µm wide, with necridia, mostly uniseriate, bloated but tapered/waisted towards heterocytes; cells barrel-shaped, rounded, squeezed, constricted at cross-walls, wider than long, 2.4–3.3 (6) µm × 1.3–1.7 µm, coarse granulated, with at least one big vacuole-like structure in each cell, blue-green	Creeping, irregular, coiled or flexuouse, 3–7 µm wide, cylindrical, not or slightly narrowed towards ends, sometimes fasciculated; monoseriate but sometimes biseriate, cells clearly constricted at cross walls, barrel shped, up to spherical 3–4 µm wide	not evaluated	not evaluated
Branches	Solitary, divaricated from basal sections			Slightly narrower than main filament	Strictly divaricated from basal sections, up to 200 µm long, uniseriate, without necridia; filaments straight, parallel; regular, not tapered towards the ends, fine granulated, fine vacuolated, cells regular, quadratic or slightly longer than wide, 2–2.3 µm × 2–2.8 µm, very slightly constricted at cross-walls, blue-green; apical tip rounded to slightly conical, yellowish, 2.8 µm × 1.8 µm;	Cells cylindrical, 2–3 µm wide and up to 4× longer than wide, terminal cells rounded	not evaluated	not evaluated
Heterocyst	Solitary, rare, 12–15 µm × 8–10, ±rectangular, rarely subglobose	Intercalary, cylindrical, 7–13 µm × 4–6 µm	Very rare, intercalary, cylindrical, 8.2 × 5.8 µm	solitary, intercalary, rarely located at the point of branching, spherical to elongated cylindrical, up to 3.5 × longer than broad, 9.0–18.5 (–26) μm × 5.5–13.8 μm	Solitary, rarely in pairs of two, thick-walled, yellow, intercalary; rectangular in branches, 2.3 µm × 3.6–5 (8.5) µm; subglobose, squeezed and boalated in basal section, 2–2.2 µm × 3.6–3.9 µm	Intercalary, cylindrical	Intercalary, rarely lateral-sessile	Intercalary, rarely lateral-sessile
Sheath	Slightly widened, not or partly slightly lamellated, initially hyaline, brownish with age	Yellow-brownish, 3–4 µm thick, stratified, marked	Hyaline, often lamellated, heavily lime-encrusted	Firm, hyaline mucilage, up to 25 μm wide, lamellated closer to the trichome	Hyaline, colorless, firm, limited, not lamellated, very tight, ruptured by the apical cells of lateral branches; wider and prominent in mature cultures	Colorless, up to yellow brown, lamellated in old filaments	not evaluated	not evaluated
Multiplication	Hormogonia, 30–35 µm × 9–10 µm	Apical hormogonia, 44–50 × 7 µm	Terminal hormogonia	not evaluated	Fragmentation of lateral branches	Long hormogonia	Hormogonia	Hormogonia

## Data Availability

Generated sequences can be found as stated under the species description.
